# Heart Failure but Not Myocardial Infarction Is Causing Bone Loss in Rodent Models in an FGF23-Independent Manner

**DOI:** 10.3390/ijms27010121

**Published:** 2025-12-22

**Authors:** Svetlana Slavic, Nejla Latic, Norbert Hassler, Stéphane Blouin, Jochen Zwerina, Reinhold G. Erben

**Affiliations:** 1 Ludwig Boltzmann Institute of Osteology, Heinrich-Collin-Strasse 30, 1140 Vienna, Austria; svetlana.slavic@lbg.ac.at (S.S.);; 2 Department of Biological Sciences and Pathobiology, University of Veterinary Medicine, 1210 Vienna, Austria; 3 1st Medical Department, Hanusch Hospital, 1140 Vienna, Austria; 4 Metabolic Bone Diseases Unit, School of Medicine, Sigmund Freud University Vienna, 1020 Vienna, Austria; 5 Health Care Center Mariahilf, Österreichische Gesundheitskasse (The Austrian Health Insurance Fund), ÖGK, 1060 Vienna, Austria

**Keywords:** myocardial infarction, heart failure, transverse aortic constriction, bone mineral density, osteoporosis, fibroblast growth factor-23

## Abstract

Myocardial infarction (MI) and heart failure (HF) are associated with low bone mineral density (BMD). We aimed to investigate whether MI and HF directly cause bone loss using three different experimental models of cardiac injury. Firstly, terminal myocardial infarction was induced in adult wild-type mice by coronary ligation, followed by peripheral quantitative computed tomography (pQCT) and histomorphometric and biochemical analyses at 4 and 9 weeks post-infarction. Secondly, myocardial ischemia–reperfusion injury (I/R) was performed in 4- and 9-month-old rats, followed by bone phenotyping 4 weeks after injury. Finally, transverse aortic constriction (TAC) was performed in adult wild-type mice, double *Fgf23*/VDR (fibroblast growth factor-23/vitamin D receptor) mutants, and VDR-deficient mice to investigate bone changes in an HF model caused by afterload-induced cardiac hypertrophy, 4 and 6 weeks after TAC. We found unchanged BMD after MI, in both the terminal ischemia model in mice and in the myocardial I/R injury model in young and aged rats. On the other hand, TAC significantly reduced especially cortical BMD in femora. Global knockout of *Fgf23* in *Fgf23*/VDR compound mutants did not rescue the TAC-induced skeletal phenotype. Collectively, our data demonstrate that TAC-induced HF, but not MI, is causing bone loss in mice in an FGF23-independent manner.

## 1. Introduction

Osteoporosis and cardiovascular conditions such as myocardial infarction (MI) and heart failure (HF) are associated with high morbidity, mortality, and health care costs. Non-cardiovascular diseases such as osteoporosis are increasingly contributing to mortality among MI survivors and HF patients [1,2,3,4]. Moreover, large-scale population-based studies demonstrate that MI and HF increase the risk of developing an osteoporotic fracture [5,6]. However, it is still unknown whether heart disease and osteoporosis are epiphenomena, or if there is a direct causal relationship between them.

During growth and adult life, bone is continuously remodeled under the influence of various endogenous stimuli and hormones. MI and HF may disturb this balance by various mechanisms, including inflammation, oxidative injury, or hormonal disturbances such as hyperparathyroidism [4,7,8]. We and others have recently shown that circulating fibroblast growth factor-23 (FGF23) levels are increased in murine MI and left ventricular hypertrophy models [9,10,11]. FGF23 may affect bone through its phosphaturic action, by suppression of active vitamin D hormone synthesis, by modulating parathyroid hormone (PTH) levels, or by a direct action on bone cells [12,13]. It is currently unknown whether FGF23 plays a role in the potential mechanistic axis between heart and bone disease.

Despite clinical evidence suggesting an association between osteoporosis and cardiac diseases, direct proof that MI or HF cause osteopenia remains scarce. To explore the relationship between heart disease and bone loss and to clarify the underlying mechanisms, experimental models of MI and HF have been employed. In contrast to the clinical situation, animal models permit studying the effects of cardiac disease on bone without the interference of confounding factors such as vitamin D deficiency, co-medications, and comorbidities. Experiments in rodent models indicate that MI may induce bone loss, but the underlying mechanism remains obscure [14,15]. In addition, a recent rat study demonstrated that pressure overload-induced HF leads to bone loss, potentially mediated by sympathetic activation [16].

To address the question of whether heart disease leads to bone loss and to further explore the pathophysiological role of FGF23, we employed well-established experimental models of MI in both mice and rats, as well as the transverse aortic constriction (TAC) model of left ventricular hypertrophy and HF in mice. To test the hypothesis that FGF23 may mediate TAC-induced bone loss, we employed *Fgf23*/vitamin D receptor (VDR) compound mutant mice [17]. Single *Fgf23* knockout mice cannot be subject to TAC due to their severe phenotype. However, *Fgf23*/VDR compound mutant mice are healthy and can be studied until old age [17]. Our data show that TAC-induced HF, but not MI, is causing bone loss in mice in a FGF23-independent manner.

## 2. Results

### 2.1. Myocardial Infarction Induced by Terminal Ischemia or Ischemia/Reperfusion Injury Does Not Cause Osteopenia in Mice or Rats

Recent data suggest a direct impact of MI on bone density in mice as soon as 9 days after MI in the C57BL/6N strain [14], while in apolipoprotein E (ApoE)-deficient mice bone loss was reported 8 weeks after MI [15]. RANKL is the key cytokine driving osteoclast maturation, and we previously showed increased cardiac RANKL expression, 4 weeks after MI in mice [18]. Therefore, we hypothesized that MI may lead to bone loss via enhanced osteoclast activity, 4 weeks after MI.

**Figure 1:**
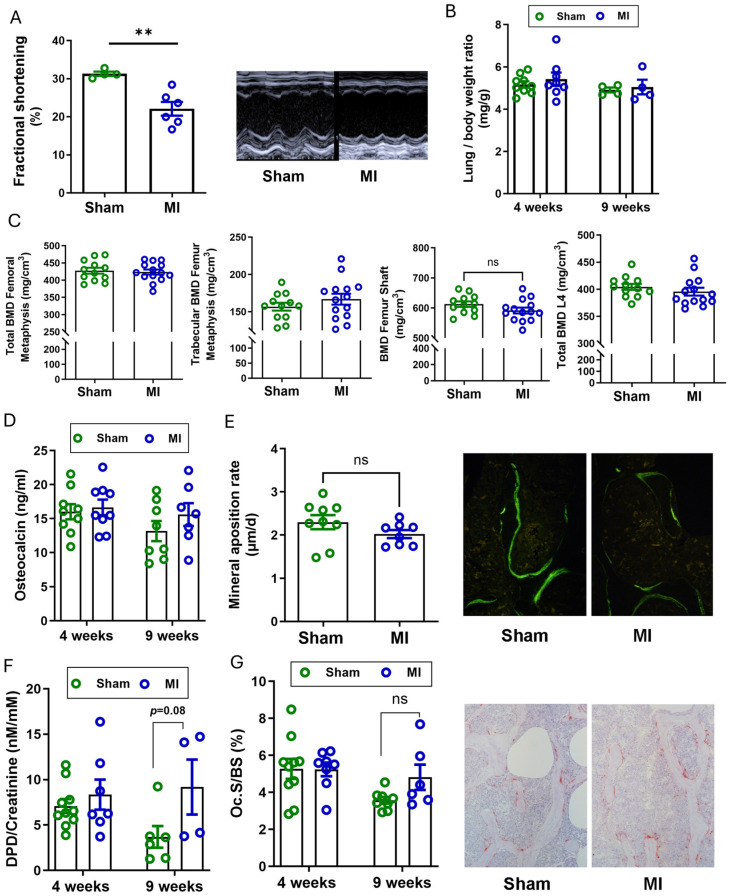
Effect of MI on BMD and bone turnover in mice, 4 weeks and 9 weeks post-surgery. (**A**) left: fractional shortening measured by echocardiography, 3 weeks post-MI, *n* = 4–6, right: representative echocardiograms of M-mode analysis after sham and MI surgery; (**B**) lung weight to body weight ratio, 4 and 9 weeks after MI, *n* = 4–9; (**C**) BMD of femora and vertebrae (L4) analyzed by pQCT in adult mice, 4 weeks post-MI (*n* = 12–14); (**D**) serum osteocalcin measured by ELISA, *n* = 7–9; (**E**) mineral apposition rate analyzed in vertebral cancellous bone (L1) by histomorphometry and representative images of fluorochrome double labelling 4 weeks after MI, *n* = 8–9; (**F**) urinary deoxypyridinoline/creatinine (DPD/Crea) excretion, *n* = 7–9; (**G**) osteoclast surface and representative images of tartrate-resistant acid phosphatase (TRAP) staining in mouse vertebrae, 4 weeks after MI, *n* = 8–9. Data are bar dot plots ± standard error of the mean (SEM), each circle represents one value, green color represents sham group and blue color represents MI group. ** *p* < 0.01 vs. sham by *t*-test.

To test this idea, we induced terminal ischemia in the left ventricle of mice as previously described [18]. To avoid potential interference with acute cardiac remodeling, we focused on the longer-term effects of MI on bone and assessed bone mineral density (BMD) and bone turnover, 4 weeks post-surgery. As expected, cardiac function had declined significantly by 4 weeks post-MI (Figure 1A). However, body weight and lung/body weight ratio as a readout of lung congestion and HF did not show changes relative to sham controls at the 4-week time point (Supplemental Figure S1A and Figure 1B). Serum electrolytes, alkaline phosphatase activity, and PTH levels remained unchanged, 4 weeks after MI (Table 1). To assess the impact of MI on volumetric BMD in the axial and appendicular skeleton, we used peripheral quantitative computed tomography (pQCT). Surprisingly, no significant changes were observed in total, trabecular, or cortical BMD in either the appendicular (femur) or the axial (L4 vertebra) skeleton at 4 weeks post-MI, relative to sham controls (Figure 1C and Supplementary Figure S1B). To capture any potential long-term effects of MI on bone, we examined an additional time point at 9 weeks post-MI. However, as shown in Figure 1B and Table 2, no significant changes in BMD were observed by pQCT at 9 weeks following MI surgery. Similar to the 4-week time point, lung oedema was absent in MI mice at 9 weeks post-surgery (Figure 1B).

To assess bone turnover, we measured biochemical bone turnover markers and performed bone histomorphometry. In line with the lacking effect of MI on BMD, the bone formation marker osteocalcin and the bone resorption marker urinary deoxypyridinoline (DPD) as well as histomorphometric analysis in the cancellous bone of the femoral metaphysis did not reveal statistically significant effects on bone turnover. At 9 weeks post-MI, we observed a non-significant trend towards higher urinary DPD excretion and osteoclast numbers (Figure 1F,G). However, these effects did not reach statistical significance.

Taken together, our results indicate that terminal myocardial ischemia per se does not affect BMD and bone turnover in mice, 4 and 9 weeks post-surgery.

**Figure 2:**
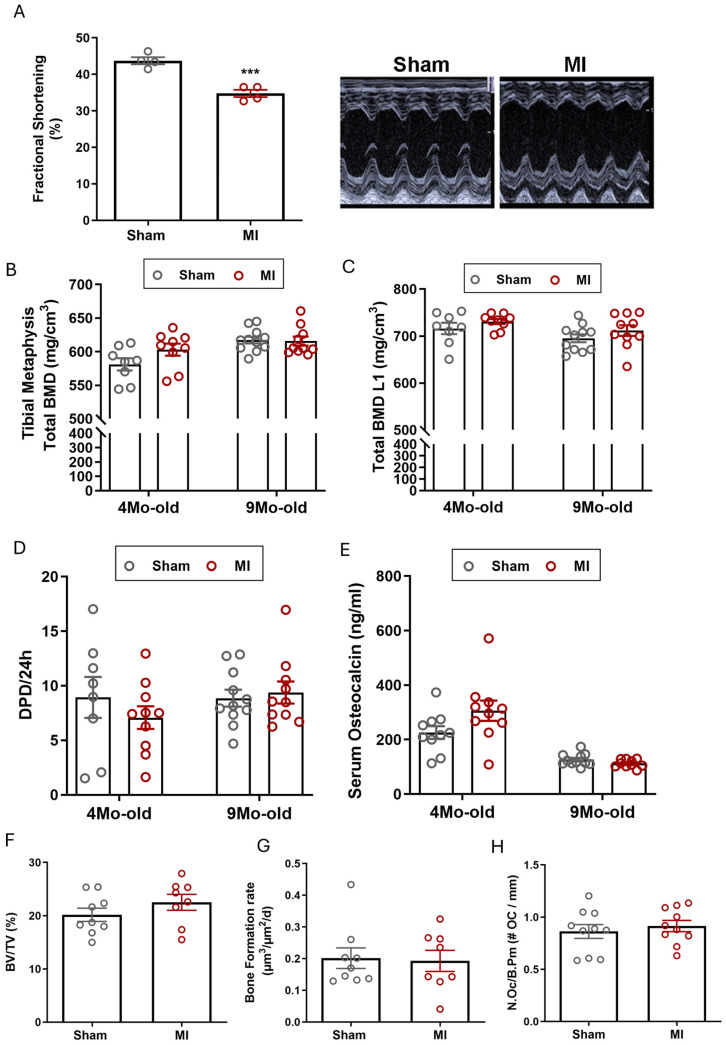
Bone phenotype after 4 weeks of cardiac ischemia/reperfusion injury in young adult (4-month-old) and aged (9-month-old) rats. (**A**) Fractional shortening analyzed by echocardiography 4 weeks post-surgery and representative M-Mode echocardiograms in 4-month-old rats (*n* = 4 per group); (**B**,**C**) bone mineral density (BMD) analyzed by pQCT in the tibial metaphysis and in the first lumbar vertebra (*n* = 8–11); (**D**) urinary deoxypyridinoline/creatinine (DPD/Crea) excretion and (**E**) serum osteocalcin levels analyzed by ELISA, *n* = 9–11; (**F**–**H**) histomorphometric analysis of vertebral cancellous bone (L2) in 4-month-old rats, 4 weeks after I/R injury: (**F**) bone volume, (**G**) bone formation rate expressed as µm^3^/µm^2^/d after fluorochrome double labelling, (**H**) number of osteoclasts per bone perimeter, *n* = 8–10. Data are bar dot plots ± SEM, each circle represents one value, gray color represents sham group and red color represents MI group. *** *p* < 0.001 vs. sham by *t*-test.

To test whether myocardial reperfusion injury and the associated oxidative stress would affect bone remodeling in a different manner than terminal ischemia, we induced ischemia–reperfusion injury (I/R) in rats, recapitulating the clinical situation where coronary artery blood flow can be restored by catheterization. The rat model was chosen because it is technically very difficult to perform cardiac I/R injury in mice. In addition, we intended to increase the robustness of our study by investigating MI-related changes in two different species. Both young, 4-month-old, and aged, 9-month-old rats were investigated, and the 4-week time point was chosen to match the mouse experiments. As shown in Figure 2A, I/R injury significantly reduced systolic cardiac function by 9%. However, total BMD quantified by pQCT at the tibial metaphysis and at the first lumbar vertebra remained unchanged in both 4- and 9-month-old rats, 4 weeks after I/R injury (Figure 2B,C). Similarly, trabecular and cortical BMD were not affected by I/R injury (trabecular BMD of tibial metaphysis in sham 240.3 ± 7.7 vs. 257.3 ± 11.67 after I/R; cortical BMD of tibial shaft 1081 ± 3.1 in sham vs. 1076 ± 6.2 after I/R, means ± SEM). The biochemical markers of bone resorption and urinary DPD excretion, and of bone formation and serum osteocalcin, remained unchanged after I/R injury in both young and aged rats (Figure 2D,E). In line with these findings, histomorphometric analysis of the second lumbar vertebra did not reveal any changes in bone volume, bone formation rate, or number of osteoclasts in 4-month-old MI rats (Figure 2F–H). Hence, similar to the mouse MI experiments using terminal ischemia, our results suggest that BMD and bone turnover are not affected by I/R injury in young adult and aged rats.

### 2.2. Pressure Overload-Induced Heart Failure Reduces Cortical Bone Mineral Density

**Figure 3:**
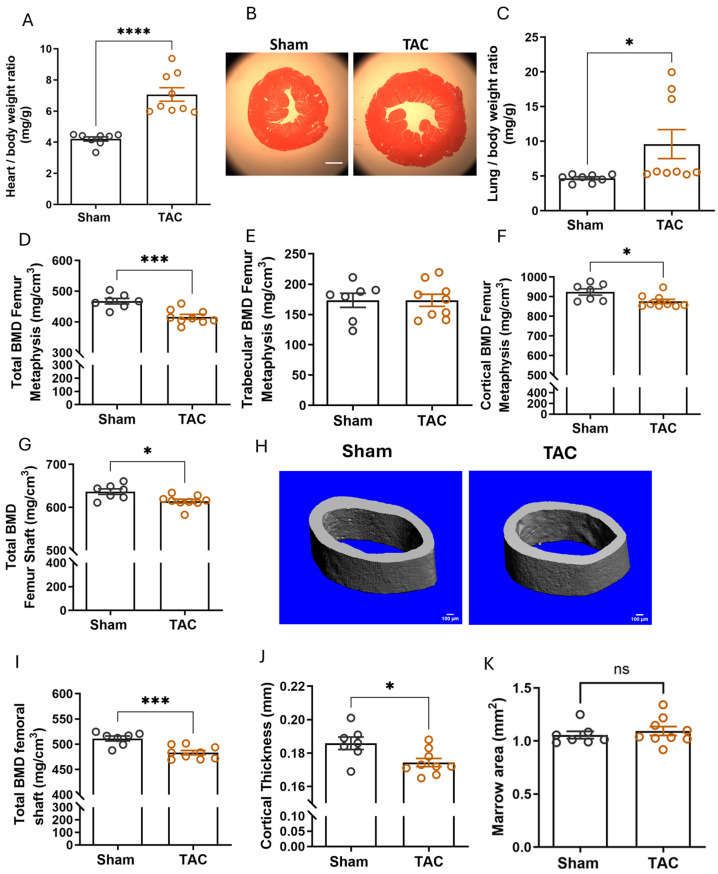
Pressure overload by transverse aortic constriction (TAC) induces osteopenia in adult WT mice. (**A**) Heart/body weight ratio is significantly increased 6 weeks after TAC; (**B**) representative H&E-stained cardiac cross-sections, 6-weeks after sham or TAC surgery (scale bar: 1000 µm); (**C**) TAC induced lung oedema 6 weeks after surgery as evidenced by increased lung/body weight ratio; (**D**–**F**) total BMD, trabecular BMD, cortical BMD analyzed by pQCT in the femoral metaphysis; (**G**) total BMD analyzed by pQCT in the femoral midshaft region; (**H**) representative µCT images of the femoral midshaft region; (**I**–**K**) µCT analysis of the femoral midshaft region: (**I**) total BMD, (**J**) cortical thickness, (**K**) marrow area. Data are bar dot plots ± SEM, each circle represents one value, gray color represents sham group and orange color represents TAC group *n* = 7–9. * *p* < 0.05 vs. sham, *** *p* < 0.001 vs. sham, **** *p* < 0.0001 vs. sham by *t*-test. ns—not significant.

The terminal or transient ischemia-induced myocardial injury models shown above did not progress to heart failure, as evidenced by unchanged lung/body weight ratio (Figure 1B). To test whether heart failure leads to changes in bone remodeling, we used TAC, a well-established model of non-ischemic heart failure by chronically increased cardiac afterload [19]. Successful constriction was confirmed by increased maximal aortic flow velocity distal to the ligation (Supplemental Figure S2A). As expected, TAC induced significant cardiac hypertrophy, impaired cardiac function, and caused lung oedema, 6 weeks post-surgery (Figure 3A–C and Supplemental Figure S2B,C). pQCT analysis revealed significantly reduced total BMD at the femoral metaphysis and the femoral shaft (Figure 3D,G). However, femoral trabecular BMD was not affected (Figure 3E). Rather, the reduced total BMD at the femoral metaphysis was caused by a reduction in cortical BMD (Figure 3F). Micro-computed tomography (µCT) analysis of the femoral shaft confirmed loss of cortical BMD and cortical thinning in TAC mice (Figure 3H–K). In agreement with the pQCT data, histomorphometric analysis of trabecular bone in the femoral metaphysis confirmed unchanged bone volume and structure (Table 3). Bone formation rate did not show differences between sham and TAC mice, but osteoclast numbers were increased relative to sham controls, 6 weeks after TAC (Table 3).

In lumbar vertebrae, on the other hand, pQCT analysis showed a non-significant trend towards reduced trabecular BMD, but unchanged cortical BMD in TAC mice (Supplemental Figure S2D–G). In agreement with this finding, bone histomorphometry confirmed the non-significant trend towards reduced bone volume and revealed trabecular thinning in vertebral cancellous bone (Table 4). However, bone formation rate and osteoclast number remained unchanged in vertebral cancellous bone of TAC mice (Table 4). Interestingly, osteoid maturation time was slightly but significantly prolonged in TAC mice (Table 4).

To gain further insight into the endocrine mechanisms that may underlie bone loss in heart failure, we analyzed serum electrolytes, biochemical bone markers, and PTH levels. Serum levels of alkaline phosphatase, calcium, phosphate, and potassium were unchanged, 6 weeks after TAC. However, we found hypernatremia in TAC mice, possibly due to hyperaldosteronism and/or increased FGF23 levels [9,20,21] (Table 5). Urinary DPD excretion, a whole-body bone resorption marker, was not significantly changed, 6 weeks after TAC (Table 5). Secondary hyperparathyroidism is common in patients with chronic heart failure and serum PTH levels correlated with reduced BMD in this patient population [4]. However, serum PTH levels in TAC mice were not changed, 6 weeks post-surgery (Table 5), suggesting that PTH is not driving the bone phenotype in mice with heart failure.

Collectively, these data show that hypertrophic cardiomyopathy and heart failure led to reduced cortical bone mass in the appendicular skeleton and a trend towards trabecular bone osteopenia in the axial skeleton of TAC mice. The osteopenia in TAC mice was associated with a site-specific, PTH-independent upregulation in osteoclastic bone resorption.

### 2.3. TAC-Induced Bone Loss Is Likely Not Caused by Hypoperfusion

**Figure 4:**
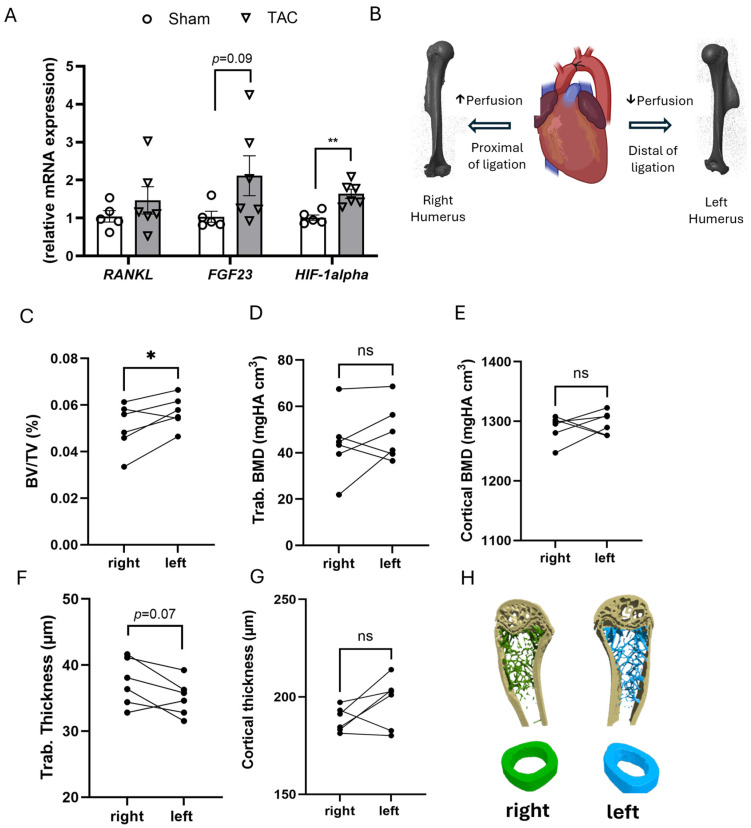
Gene expression analysis in the L5 vertebra and analysis of the effect of hypoperfusion on left and right humeri by µCT. (**A**) Relative bone *RANKL*, *Fgf23*, and *HIF-1alpha* mRNA expression in the L5 vertebra, 4 weeks after TAC, *n* = 5–6, data are presented as bar dot plots ± SEM, circles and white bars are sham, triangle and grey bars are TAC, ** *p* < 0.01 vs. sham by non-paired *t*-test. (**B**) Schematic illustration of the experimental hypothesis: aortic ligation (TAC) creates a pressure gradient, leading to differential perfusion of the right and left humerus. TAC image created in BioRender.com, humeri are 3D reconstructions from µCT images; (**C**–**G**) µCT analysis of the right and left humerus, 4 weeks after TAC, each point represents a single value; *n* = 6 per group; (**H**) 3D rendering from µCT images of a left and right humerus from the same TAC mouse. * *p* < 0.05 by paired *t*-test; ns—not significant.

To further characterize the local changes in gene expression in bone after TAC, we measured mRNA expression of *RANKL*, *Fgf23*, and *HIF-1alpha* (*hypoxia-inducible factor 1- alpha*) in the fifth lumbar vertebra (L5), 4 weeks after TAC. In line with unchanged osteoclast numbers seen in histomorphometry, mRNA expression of *RANKL* was not significantly changed in L5 vertebrae, 4 weeks after TAC (Figure 4A). *Fgf23* mRNA abundance tended to be increased, but this did not reach statistical significance (Figure 4A). However, the expression of *Hif-1alpha* was significantly increased, 4 weeks after TAC as compared to sham (Figure 4A).

Transcription of *HIF-1alpha* is induced by tissue hypoxia [22]. Thus, we hypothesized that reduced bone perfusion may be an important mechanism of bone loss in heart failure. In our TAC model, the aortic constriction is placed between the right brachiocephalic and the left common carotid arteries (Figure 4B). Hence, the right brachiocephalic artery is upstream of the constriction site, whereas the left subclavial artery is downstream of the constriction site. Blood pressure upstream of the ligation is increased in this model, whereas hypotension is found downstream of the constriction site [9,21,23]. Therefore, although this has never been measured to our knowledge, TAC should lead to reduced perfusion in the left upper extremity as compared to the right upper extremity. To test the hypothesis that hypoperfusion is underlying the TAC-induced osteopenia, we performed a comparative analysis of the right and left humerus by µCT, 4 weeks after TAC. Contrary to our hypothesis, we found slightly increased trabecular bone mass in the metaphysis of the (potentially hypoperfused) left humerus as compared to the right side. Cortical bone thickness and cortical BMD in the shaft of the humerus did not show left–right differences (Figure 4C–G). Thus, regional differences in perfusion are likely not the cause for the reduced BMD observed after TAC.

### 2.4. FGF23 Lacks Essential Role in TAC-Induced Osteopenia

We have recently shown that TAC leads to increased serum FGF23, mainly through upregulated bony FGF23 production [21]. Moreover, we reported that FGF23 impairs bone mineralization by suppressing transcription of tissue nonspecific alkaline phosphatase (TNAP) [13,24]. In line with the hypothesis that FGF23 may be involved in the pathogenesis of TAC-induced osteopenia, we observed an increase in osteoid maturation time in vertebral cancellous bone of TAC mice (Table 4). To assess whether increased FGF23 is responsible for the reduced BMD after heart failure, TAC was induced in mice with a global deletion of *Fgf23*. Because the early lethality of *Fgf23* deletion is prevented by genetic disruption of vitamin D signaling (VDR^Δ/Δ^), TAC was induced in double-mutant *Fgf23*^−/−^/VDR^Δ/Δ^ mice. Wild-type (WT) and VDR^Δ/Δ^ mice served as controls. We previously reported that VDR deficiency does not affect the TAC-induced increase in serum FGF23 [9]. To prevent hypocalcemia and hypophosphatemia in VDR-deficient mice, all animals in this experiment were kept on a diet enriched with calcium, phosphorus, and lactose.

**Figure 5:**
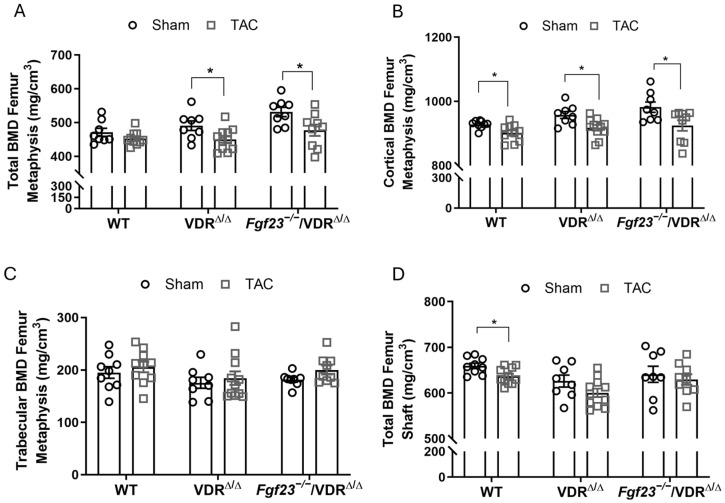
*Fgf23* deficiency does not rescue TAC-induced bone loss. (**A**–**D**) pQCT analysis of femora from wildtype (WT), VDR^Δ/Δ^ mice, and *Fgf23*^−/−^/VDR^Δ/Δ^ mice on a calcium- and phosphorus-enriched rescue diet, 4 weeks after TAC. Data are bar dot plots ± SEM, black circles represent sham animals, grey squares represent TAC animals, *n* = 8–11. * *p* < 0.05 vs. sham of the same genotype by *t*-test.

pQCT analysis of the femoral metaphysis revealed a significant reduction in total and cortical BMD in VDR^Δ/Δ^ and *Fgf23*^−/−^/VDR^Δ/Δ^ mice, 4 weeks after TAC (Figure 5A,B). In line with the data shown above, trabecular BMD at the femoral metaphysis was not changed after TAC in any of the genotypes (Figure 5C). pQCT analysis of the femoral shaft showed reduced total BMD and cortical thinning in WT mice after TAC, and similar trends in *Fgf23*^−/−^/VDR^Δ/Δ^ mice and VDR^Δ/Δ^ controls, although the latter effect did not reach statistical significance (Figure 5D). These data suggest that excessive FGF23 is not the major driving factor behind the TAC-induced reduction in bone mass after heart failure. It is interesting to note in this context that the high-calcium diet fed to all mice in this experiment did not prevent the bone loss observed in WT mice (Figure 5B,D).

## 3. Discussion

Our data demonstrate that heart failure resulting from pressure overload-induced left ventricular hypertrophy in mice leads to bone loss, a phenomenon that we did not observe in rodent models of myocardial infarction. HF-induced bone loss primarily affected the cortical bone, and both pQCT and histological analyses suggested that the vertebrae may be affected differently than the femur. Although our data do not provide a final mechanistic explanation for these observations, we could exclude PTH and FGF23 as hormonal mediators of HF-induced osteopenia. Also, HF-induced bone mineral loss could not be prevented by a high-calcium diet.

### 3.1. Exploring the Heart–Bone Axis: Experimental Evidence and Clinical Implications

A cross-sectional study demonstrated that male patients who self-reported previous MI were at greater risk of having a low bone mass [25]. This risk increase of about 28% was independent of risk factors common for both MI and osteoporosis, such as age, smoking, physical activity, and BMI [25]. Longitudinal investigations of BMD in patients after MI are lacking, and due to the cross-sectional study design of previous studies, it is difficult to examine cause–effect relationships [26]. Vice versa, several studies reported an increased risk of cardiovascular events in patients with established osteoporosis [27,28]. Interestingly, among the patients at high or imminent risk for osteoporotic fracture, the major adverse cardiovascular event (MACE) rate was the highest in patients with incident fragility fracture [27]. However, it remains unknown whether common pathophysiological determinants link the development of these diseases as co-morbidities, or whether cardiovascular diseases indeed cause osteopenia/osteoporosis [26].

In contrast to previously published studies [14,15], we could not demonstrate significant bone loss following the induction of transient or terminal myocardial ischemia in rats and mice. However, the methodology used in our study differs substantially from the work of Tjandra et al. [14]. Firstly, we did not perform repeated measurements of BMD before and after MI. Rather, we used sham-operated animals as a control group. Opening of the thorax and of the pericardium is a major stressor and activates the sympathetic nervous system, potentially leading to post-operative pain, reduced well-being, and decreased mobility, despite analgesia. Hence, although longitudinal measurement of BMD before and after MI is intuitive and statistically advantageous, comparing unoperated and operated animals in a surgical MI model may be potentially misleading. In agreement with this scenario, a post-operative loss of lean body mass was reported in MI animals in the study by Tjandra et al. [14]. Secondly, Tjandra et al. [14] measured BMD 9 days post-MI, whereas our study focused on the long-term effects of MI on the bone over 9 weeks. Thus, the effect of MI on BMD could be transient, as other experiments of the same group have shown [29]. Interestingly, a study performed in ApoE-deficient mice demonstrated that MI-induced bone loss could be prevented by exercise [15].

Although we were unable to confirm MI-induced bone loss in our study, we found bone loss in mice with TAC-induced myocardial hypertrophy and heart failure. This is in line with data reported by Guan et al. [16]. The latter authors performed their study in rats, using a different TAC model, where the abdominal and not the ascending aorta was ligated. They found significant loss of both trabecular and cortical bone in tibial and vertebral sites [16]. Our study in mice indicates a predominant involvement of cortical bone. Previous research has shown that cortical bone is the main contributor to femoral neck strength [30]. Furthermore, a recent meta-analysis reported that heart failure increases the risk of hip fractures with a hazard ratio of 2.2, whereas the hazard ratio for any fracture was only 1.67, highlighting the critical role of cortical bone [31]. So far, only few national osteoporosis guidelines regard heart failure as a risk factor for osteoporotic fracture [32,33].

### 3.2. Pathophysiological Mechanisms Linking Heart Failure to Bone Loss

The mechanisms by which heart failure leads to reduced BMD and increased fracture risk have not been fully elucidated. Since loss of BMD is reproducible in animal models of heart failure, increased fracture risk in HF patients cannot be explained solely by common risk factors, effect of co-medication, or reduced sun exposure due to reduced mobility.

#### 3.2.1. Role of Sympathetic Activation

It is well established that the sympathetic nervous system is activated in congestive heart failure [34], leading to increased sympathetic outflow not only in the heart but also peripheral organs such as kidneys and skeletal muscles, as described in a recent review [35]. Beta-adrenergic receptors regulate osteoblastogenesis and RANKL expression [36], and an unselective beta receptor blockade using propranolol mitigates the deleterious skeletal effects of ovariectomy in mice [37]. Furthermore, elevated norepinephrine levels have been detected in cortical bone chips of mice during lactation, following glucocorticoid treatment, and after ovariectomy [38]. Interestingly, osteocyte-specific deletion of *adrenoceptor beta 2* (*Adrb2*) reduced osteocyte-mediated cortical bone resorption in lactating mice [38]. To our knowledge, no clinical or experimental studies have directly demonstrated increased sympathetic activity within the bone in heart failure. However, indirect evidence has been provided by the study of Guan et al., who demonstrated upregulation of β1 and β2 adrenergic receptor expression in tibial bone after TAC [16]. In addition, the latter authors showed that BMD and bone structure was improved by chemical sympathectomy using guanethidine in the TAC model [16]. Clinical data from the Framingham Osteoporosis Study showed that women on beta-blockers had a 3.7% increase in femoral neck BMD as compared with nonusers, and this effect was partly dose-dependent [39]. Studies specifically investigating the effect of beta blocker use on fracture risk in patients with heart failure are lacking. However, existing evidence from cardiovascular or general adult populations, according to a recent meta-analysis of observational studies, suggests that beta blocker use has, if any, only a modest effect on fracture risk reduction [40,41]. Thus, it is likely that additional mechanisms are involved.

#### 3.2.2. Role of RANKL and HIF-1 α Signaling

RANKL signaling is essentially involved in bone remodeling by promoting osteoclastogenesis and osteoclast activity. The abundance of RANKL as well the RANKL/OPG ratio is significantly increased in the bone marrow plasma of patients with heart failure as compared to patients with preserved systolic and diastolic cardiac function diagnosed with coronary artery disease [42]. Several signaling pathways, including inflammatory mediators [43], PTH [44], vitamin D [45], and WNT-signaling [46], are major upstream pathways that regulate RANKL expression in general, but little is known which signaling pathway activates RANKL production in heart failure. In the study by Leistner et al., PTH did not correlate with RANKL expression in patients with HF [42]. Guan et al. showed that sympathectomy reduced *RANKL* transcription in their experimental rat model of HF [16]. In our study, we did not find a significant upregulation of *RANKL* gene expression in vertebrae after TAC. However, we for the first time report increased bony expression of *HIF-1alpha* in TAC mice, 4 weeks post-surgery. By targeting osteoblasts, osteoclasts, and vascular cells, HIF-1alpha plays an important role in the regulation of bone homeostasis [22]. HIF-signaling also drives, directly or indirectly, the expression of FGF23 in bone [47,48]. Further research is needed to confirm our findings and to elucidate the potential role of HIF-1α in TAC-induced heart failure. Specifically, it remains unclear whether HIF signaling mediates bone loss or serves as a bone-protective, compensatory mechanism in heart failure.

#### 3.2.3. Role of Hypoperfusion

Based on our finding of increased *HIF-1alpha* in bones of TAC mice, we hypothesized that hypoperfusion may be a key mechanism mediating bone loss in HF. It was previously shown that aortic constriction in the mouse TAC model increases flow velocities in the right carotid artery as compared to the left [23]. In agreement with these findings, we previously measured increased aortic blood pressure upstream of the constriction site in TAC relative to sham mice [9]. Thus, we reasoned that the difference in perfusion between the left and right upper extremities could be leveraged to test the hypothesis that hypoperfusion may mediate HF-induced bone loss. However, contrary to this hypothesis, trabecular bone volume was indeed increased in the left humerus as compared to the right, and there was no left–right difference in cortical bone. Based on these results, it is unlikely that HF-induced bone loss is mainly induced by hypoperfusion of bones. A potential caveat in this reasoning is that, due to Bernoulli’s law, the aortic blood velocity distal from the ligation is actually increased, as shown in Supplemental Figure S2A. Thus, more sophisticated methods such as contrast-enhanced ultrasound (CEUS) or in vivo dynamic contrast-enhanced MRI assessment may be needed to better understand the role of bone perfusion in experimental models of heart failure.

#### 3.2.4. Role of FGF23

In our study, we showed that osteoid maturation time in vertebral cancellous bone was increased after TAC, and we hypothesized that this may be FGF23-mediated. FGF23 is an osteocyte-derived phosphaturic hormone that suppresses the synthesis of 1,25-dihydroxyvitamin D (calcitriol), ultimately leading to hypophosphatemia and impaired bone mineralization in patients with disorders characterized by elevated FGF23 secretion, such as tumor-induced osteomalacia (TIO) or X-linked hypophosphatemia (XLH). We earlier demonstrated that FGF23 suppresses the expression of tissue-nonspecific alkaline phosphatase (TNAP) in bone cells, resulting in the accumulation of the mineralization inhibitor pyrophosphate [24]. In addition, we found significantly elevated circulating FGF23 levels in TAC mice in several studies [9,21]. Data from heart failure patients also show elevated FGF23 levels in serum and in bone marrow plasma [49]. However, existing data from the general population are inconsistent concerning the effect of FGF23 on BMD. Several studies, including Mendelian randomization and cross-sectional studies suggest a negative correlation between FGF23 and BMD [50,51,52,53]. However, a prospective study in a cohort of community-dwelling, well-functioning older adults found no significant association between FGF23 levels and BMD or fracture risk over a median follow-up of 4.95 years [54]. Consistent with these findings, our data do not support a significant role of FGF23 in heart failure-induced osteopenia.

In conclusion, our study showed that TAC-induced heart failure leads to cortical bone osteopenia in a PTH- and FGF23-independent manner in mice. Together with available evidence from clinical studies and other preclinical studies, our data support the inclusion of bone health and fracture risk assessment as part of heart failure management.

### 3.3. Limitations of the Study

The current study has several limitations. First, it was designed as a purely preclinical experimental study and therefore lacks human data. Second, the duration of MI experiments in mice was limited to 9 weeks, and the I/R experiment in rats was limited to 4 weeks. Animals after MI and I/R injury did not develop heart failure; thus we cannot rule out the possibility that a longer observation period following cardiac injury might have revealed an effect on BMD. Third, the results of qRT-PCR analysis may be biased because we used only a single housekeeping gene, *ornithine decarboxylase antizyme 1* (*Oaz1*), for normalization. Future experiments should especially re-assess the bony expression of *HIF-1alpha* in TAC-induced bone loss, using two or more housekeeping genes for normalization. Finally, we did not directly assess differences in bone perfusion between the left and right humerus in the TAC model. Further studies should include advanced imaging techniques, such as CEUS and dynamic contrast-enhanced MRI, to investigate whether hypoperfusion of the bone mediates bone loss in heart failure.

## 4. Materials and Methods

### 4.1. Ethics Statement

All animal procedures were approved by the Animal Welfare Committee of the Austrian Federal Ministry of Education, Science and Research and were undertaken in accordance with prevailing guidelines for animal care and welfare (permit numbers BMWF-68.205/0111-II/10b/2010, originally approved 30 April 2010, and BMWF-68.205/0153-WF/V/3b/2014, originally approved 13 October 2014).

### 4.2. Animals

All animals were kept in groups of 2–5 mice and 2–5 rats at 22–24 °C and a 12 h light/12 h dark cycle with free access to tap water and a commercial rodent diet (Sniff™, Soest, Germany). To measure the rate of bone formation in mouse experiments, animals were injected with calcein (20 mg/kg in 1.4% NaHCO_3_) 4 days and 2 days before necropsy. To investigate the role of FGF23 signaling in HF-induced bone loss, groups of adult male WT mice and of mice expressing a non-functioning vitamin D receptor (VDR^Δ/Δ^) and compound mutants deficient in VDR and FGF23 (*Fgf23*^−/−^/VDR^Δ/Δ^) were used. WT, VDR^Δ/Δ^, and *Fgf23*^−/−^/VDR^Δ/Δ^ mice were maintained on a so-called rescue diet (Sniff™) enriched in calcium (2.0%), phosphorus (1.25%), and lactose (20%) to normalize mineral homeostasis in VDR-ablated mice [55]. Genotyping of the mice was performed as described [9].

### 4.3. Myocardial Infarction in Mice

Terminal myocardial ischemia was induced in 4-month-old male C57BL/6N mice as previously described [10]. Mice were anaesthetized with ketamine/medetomidine (100/0.25 mg/kg i.p.) anesthesia. Endotracheal intubation was performed after disappearance of the paw pinch reflex. Animals were ventilated with a tidal volume of 200 μL and a frequency of 210 breathing cycles per min using a small animal ventilator (MiniVentTyp 845, Hugo Sachs Elektronik-Harvard Apparatus GmbH, March, Germany). Permanent ligation of the left descending coronary artery was performed after a left lateral thoracotomy. Analgesic (buprenorphine 0.25 mg/kg s.c.) and antibiotic (enrofloxacin, 10 mg/kg s.c.) were injected for 4 and 5 days, respectively. Sham animals underwent the same procedure but without the arterial ligation.

Mice were killed 4 and 9 weeks after myocardial infarction by exsanguination from the abdominal vena cava under ketamine/xylazine anesthesia (70/7 mg/kg i.p.). Serum and tissue samples were flash frozen and stored at −80 °C until assayed, or processed for histological analysis.

### 4.4. Myocardial Ischemia/Reperfusion Injury in Rats

Briefly, I/R injury was performed under general anesthesia with medetomidine/fentanyl/midazolam (150 µg/kg/5 µg/kg/2 mg/kg i.p.) by ligating the left descending coronary artery for 30 min followed by reperfusion under controlled ventilation with 100% oxygen [56]. Anesthesia was antagonized with Atipamezol/Flumazenil/Naloxon (0.75/0.2/0.12 mg/kg) at the end of surgery. Pain was managed by metamizole (100 mg/kg s.c., two doses at 6 h interval on the surgery day) and continued with carprofen treatment (5 mg/kg s.c.) for the following 3 days. Antibiotic (enrofloxacin 10 mg/kg s.c.) was given for 5 days starting from the day of operation. Sham-operated animals underwent the same surgical procedure except for coronary ligation. Rats were euthanized 4 weeks after MI or sham surgery by exsanguination from the abdominal aorta under ketamine/xylazine anesthesia (50/10 mg/kg i.p.). Serum and tissue samples were flash frozen and stored at −80 °C until assayed, or processed for histological analysis.

### 4.5. Transverse Aortic Constriction

Transverse aortic constriction (TAC) was performed as previously described [9]. Briefly, in 4-month-old male C57BL/6 mice sternotomy was performed under general anesthesia (ketamine/medetomidine 100/0.25 mg/kg i.p.) and endotracheal ventilation. Aortic ligation was placed between the origins of the brachiocephalic and left common carotid arteries around a 27-gauge needle, using a 6–0 silk suture, followed by prompt removal of the needle. Sham animals underwent the same procedure without the aortic ligation. Post-operative medication was performed as described above for MI. Animals were killed 4–6 weeks after TAC or sham surgery.

### 4.6. Transthoracic Doppler Echocardiography

Echocardiography was performed 3 weeks after sham/MI surgery and 4 weeks after sham/TAC surgery using a 14 MHz linear-array transducer (Acuson s2000tm, Siemens, Munich, Germany) under 1% isoflurane anesthesia. Left ventricular (LV) wall thickness, internal dimensions, and fractional shortening were evaluated in anatomic M-mode recorded in the short axis view at the papillary muscles level. A minimum of 5 cardiac cycles were averaged for each parameter.

### 4.7. Serum and Urine Biochemistry

Serum alkaline phosphatase, creatinine, sodium, calcium, potassium, and phosphorous were analyzed using a Cobas c111 analyzer (Roche, Mannheim, Germany). Serum osteocalcin (Biomedical Technologies, Stoughton, MA, USA), serum parathyroid hormone (Immutopics, San Clemente, CA, USA), and total deoxypyridinoline (DPD, Metra Biosystems, Quidel Corporation, San Diego, CA, USA) concentrations in urine were assessed by commercially available ELISAs.

### 4.8. Peripheral Quantitative Computed Tomography (pQCT)

Bone specimens were collected and stored in 70% ethanol until analysis. Volumetric bone mineral density was assessed using an XCT Research M+ pQCT device (Stratec Medizintechik, Pforzheim, Germany).

### 4.9. Micro Computed Tomography (µCT)

Mouse femora and humeri were collected and stored in 70% ethanol until analysis. Quantitative micro-computed tomography of femora (µCT35, SCANCO Medical AG, Brüttisellen, Switzerland) was used to assess morphology and cortical BMD of the femoral shaft as described previously [13], using a voxel size of 5 µm (isotropic). The humeral metaphysis and shafts were measured on the µCT50 with 55 kV and 82 µA with 750 ms integration time, 0.18° rotation step, and a 5 µm voxel size. Dragonfly 3D World (Version 2025.1, Comet Technologies Canada Inc., Montreal, QC, Canada, https://dragonfly.comet.tech/) was used to perform the segmentation with an AI-assisted deep learning model and 3D rendering. The plugin BoneJ for ImageJ (1.54 d) was used to assess the trabecular microarchitecture and trabecular and cortical BMD. The μCT measurements were performed in compliance with previously published guidelines [57].

### 4.10. Bone Histology and Histomorphometry

Bones for histomorphometric analysis were fixed in 4% paraformaldehyde (PFA) overnight at 4 °C. The tissue specimens were dehydrated and embedded in a methylmethacrylate (MMA) embedding mixture. Histomorphometry of the cancellous bone (proximal tibial metaphysis and L1) was performed using OsteoMeasure 3.0 (OsteoMetrics, Decatur, GA, USA) and AxioVision 4.6 (C. Zeiss, Oberkochen, Germany) software for image analysis as described previously [58].

### 4.11. RNA Isolation and Quantitative RT-PCR

Fifth lumbar vertebrae (L5) from adult WT mice, 4 weeks after TAC, were defleshed and shock-frozen in liquid nitrogen. Total RNA was isolated after homogenization using TRI Reagent^®^ Solution (Invitrogen, Waltham, MA, USA). The concentration and purity of isolated RNA were determined spectrophotometrically (NanoDrop 2000; ThermoScientific, Waltham, MA, USA). An amount of 1 µg of RNA was reverse transcribed (High-Capacity cDNA Reverse Transcription Kit; Applied Biosciences, Waltham, MA, USA). Quantitative RT-PCR was performed on a Vii7 device (Applied Biosystems^®^, Foster City, CA, USA) using the 5 x Hot Firepol^®^ Eva Green kit (Solis Biodyne, Tartu, Estonia). To exclude amplification of genomic DNA, primers were designed as exon spanning, and their sequence is listed in the Supplemental Material (Supplementary Table S1). A product melting curve analysis was performed to exclude primer dimerization and nonspecific amplification. All samples were measured in duplicate and expression values were normalized to *ornithine decarboxylase antizyme 1* (*Oaz1*) mRNA.

### 4.12. Statistics

Data are generally presented as bar dot plots ± SEM in the figures, and as mean ± SEM in the tables. Statistical analysis was performed using GraphPad Prism 10. Comparisons between two groups were performed using a two-sided, unpaired *t*-test. For left–right comparisons between humeri of the same animal (Figure 4), a two-sided, paired *t*-test was used. *p* values of less than 0.05 were considered statistically significant.

## Figures and Tables

**Table 1:** Serum parameters measured 4 weeks after MI in mice.

Serum Parameter	Sham (*n* = 12)	MI (*n* = 12)	*p* Value
Alkaline Phosphatase (U/L)	46.6 ± 3.3	50.1 ± 2.7	0.41
Na (mmol/L)	150.3 ± 0.6	151.1 ± 4.6	0.86
Ca (mmol/L)	2.10 ± 0.03	2.24 ± 0.07	0.09
P (mmol/L)	3.42 ± 0.2	3.67 ± 0.3	0.48
K (mmol/L)	4.93 ± 0.4	5.93 ± 1	0.38
PTH ^a^ (pg/mL)	134.0 ± 18.4	149.8 ± 25.07	0.65

^a^ PTH, parathyroid hormone, *n* = 3 sham, *n* = 4 MI.

**Table 2:** pQCT measurements in femora and L4 vertebrae, 9 weeks after MI in mice.

Parameter	Sham (*n* = 8)	MI (*n* = 7)	*p* Value
Femoral metaphysis total BMD (mg/cm^3^)	451.9 ± 4.4	440.5 ± 6.8	0.17
Femoral metaphysis trab. BMD (mg/cm^3^)	154.1 ± 7.4	151.3 ± 7.8	0.8
Femoral shaft total BMD (mg/cm^3^)	651.9 ± 6.3	644.2 ± 9.0	0.5
L4 total BMD (mg/cm^3^)	400.6 ± 7.3	403.5 ± 4.1	0.7
L4 trabecular BMD (mg/cm^3^)	239.9 ± 6.1	242.1 ± 4.3	0.8
L4 cortical BMD (mg/cm^3^)	516.2 ± 4.6	523.2 ± 4.6	0.3

**Table 3:** Histomorphometric analysis of mouse femoral trabecular bone, 6 weeks after TAC.

Parameter	Sham (*n* = 7)	TAC (*n* = 9)	*p* Value
Bone volume (%)	4.25 ± 0.55	3.81 ± 0.38	0.51
Trabecular Thickness (µm)	26.9 ± 1.1	27.9 ± 0.7	0.46
Trabecular Separation (µm)	652.9 ± 68.3	763.6 ± 84.6	0.35
MAR (µm/day)	1.35 ± 0.14	1.53 ± 0.09	0.28
BFR/BS (µm^3^/µm^2^/d)	0.055 ± 0.013	0.050 ± 0.008	0.74
N.Oc/B.Pm (#/mm)	0.36 ± 0.06	0.63 ± 0.08 *	0.03
Osteoid Width (µm)	2.13 ± 0.19	2.54 ± 0.17	0.12
Osteoid maturation time (days)	1.74 ± 0.28	1.72 ± 0.17	0.96

* MAR, mineral apposition rate; BFR/BS, bone formation rate per bone surface; N.Oc/B.Pm, number of osteoclasts per bone perimeter.

**Table 4:** Histomorphometric analysis of mouse vertebral (L1) trabecular bone, 6 weeks after TAC.

Parameter	Sham (*n* = 5–7)	TAC (*n* = 6–9)	*p* Value
Bone volume (%)	25.9 ± 2.2	21.4 ± 1.2	0.07
Trabecular Thickness (µm)	49.61 ± 2.2	42.83 ± 1.7	0.03
Trabecular Separation (µm)	145.6 ± 9.6	158.6 ± 6.1	0.25
MAR (µm/day)	1.75 ± 0.13	1.46 ± 0.05	0.05
BFR/BS (µm^3^/µm^2^/d)	0.033 ± 0.008	0.024 ± 0.012	0.57
N.Oc/B.Pm (#/mm)	0.99 ± 0.09	1.16 ± 0.18	0.45
Osteoid Width (µm)	2.54 ± 0.17	2.45 ± 0.14	0.69
Osteoid maturation time (days)	1.41 ± 0.11	1.74 ± 0.09	0.04

**Table 5:** Biochemical parameters measured 6 weeks after TAC.

Parameter	Sham (*n* = 7–12)	TAC (*n* = 11)	*p* Value
Alkaline Phosphatase (U/L)	53.13 ± 2.52	61.02 ± 3.27	0.65
Na (mmol/L)	148.3 ± 0.81	151.3 ± 0. 87	0.02
Ca (mmol/L)	2.27 ± 0.03	2.29 ± 0.04	0.67
P (mmol/L)	2.66 ± 0.18	3.13 ± 0.18	0.08
K (mmol/L)	4.39 ± 0.26	3.97 ± 0.19	0.22
Urinary DPD/Crea (nM/mM) ^a^	7.62 ± 1.36	10.31 ± 1.67	0.25
PTH (pg/mL) ^b^	110 ± 28.4	112.3 ± 28.4	0.95

^a^ Urinary deoxypyridinoline normalized to urinary creatinine levels, *n* = 7 sham, *n* = 9 TAC. ^b^ PTH, parathyroid hormone, *n* = 6 sham, *n* = 6 TAC.

## Data Availability

The original contributions presented in this study are included in the article/Supplementary Material. Further inquiries can be directed to the corresponding author.

## Supplementary Materials

The following supporting information can be downloaded at: https://www.mdpi.com/article/10.3390/ijms27010121/s1.

## Abbreviations

The following abbreviations are used in this manuscript:

MI	Myocardial Infarction
HF	Heart Failure
BMD	Bone Mineral Density
pQCT	peripheral Quantitative Computed Tomography
µCT	Micro-Computed Tomography
I/R	Ischemia–Reperfusion
TAC	Transverse Aortic Constriction
FGF23	Fibroblast Growth Factor-23
VDR	Vitamin D Receptor
DPD	Deoxypyridinoline
HIF-1	Hypoxia-Inducible Factor 1
