# Supplementary material for: Heart Failure but Not Myocardial Infarction Is Causing Bone Loss in Rodent Models in an FGF23-Independent Manner
Source: Int J Mol Sci. 2025 Dec 22;27(1):121. doi: 10.3390/ijms27010121 (PMC12785340; doi:10.3390/ijms27010121)
Supplement: Supplementary file 1 [file ijms-27-00121-s001.zip › ijms-4017301-supplementary.pdf]

Supplemental Material

Heart failure but not myocardial infarction is causing bone loss in rodent models in an FGF23-independent manner

Svetlana Slavic [1,2,3,4,5,], Nejla Latic [2], Norbert Hassler[1], Stéphane Blouin[1], Jochen Zwerina[1,3] and Reinhold G. Erben [1,2*]

1 Ludwig Boltzmann Institute of Osteology, Heinrich-Collin-Strasse 30, 1140 Vienna, Austria;
2 Department of Biological Sciences and Pathobiology, University of Veterinary Medicine, 1210 Vienna, Austria;
3 1st Medical Department, Hanusch Hospital, 1140 Vienna, Austria;
4 Metabolic Bone Diseases Unit, School of Medicine, Sigmund Freud University Vienna, 1020 Vienna, Austria;
5 Health Care Center Mariahilf, ÖGK;
* Correspondence: reinhold.erben@lbg.ac.at

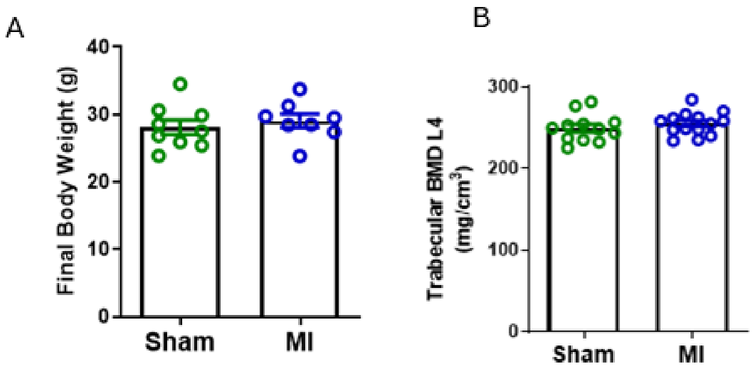

Supplementary Figure S1. *(A)* Final body weight 4 weeks after sham or MI in mice, *(B)* trabecular BMD in L4 analysed by pQCT in adult mice, 4 weeks post-surgery. Data are bar dot plots ± SEM, (n=8-14).

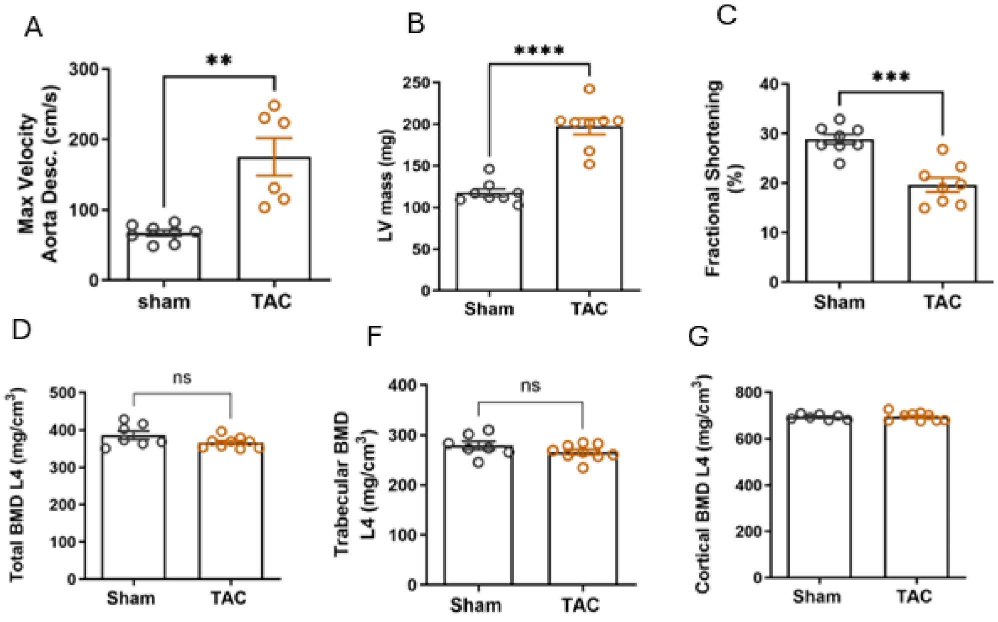

Supplementary Figure S2. *(A-C)* Analysis of blood flow velocity in the descending aorta and left ventricular (LV) structure and function by echocardiography, 4 weeks after transverse aortic constriction (TAC) in mice, *(D-G)* total BMD, trabecular BMD and cortical BMD analysed by pQCT in the lumbar vertebra L4, 6-weeks after sham or TAC surgery. Data are bar dot plots ± SEM, n=7-9, ***p < 0.001 vs. sham by t-test; ns, non-significant.

Supplementary Table S1. Mouse primer sequences for quantitative real-time PCR analysis.

Gene	Forward (5′-3′)	Reverse (5′-3′)
Hif1a	gaggctcaccatcagttatttacgt	gtgccttcatctcatcttcactgt
RANKL	tggaaggctcatggttggat	gatggtgaggtgtgcaaatgg
FGF23	tgctagggacctgccttagact	gctctagcagtgcccaagc
